# Retrospective Cohort Study of Shear-Wave Elastography and Computed Tomography Enterography in Crohn’s Disease

**DOI:** 10.3390/diagnostics13111980

**Published:** 2023-06-05

**Authors:** Minping Zhang, Enhua Xiao, Minghui Liu, Xilong Mei, Yinghuan Dai

**Affiliations:** 1 Department of Radiology, The Second Xiangya Hospital of Central South University, Changsha 410011, China; minpingzhang@csu.edu.cn (M.Z.);; 2 Department of Ultrasound Diagnosis, The Second Xiangya Hospital of Central South University, Changsha 410011, China; 3 Department of Pathology, The Second Xiangya Hospital of Central South University, Changsha 410011, China

**Keywords:** inflammatory bowel disease, Crohn’s disease, elasticity imaging techniques, computed tomography enterography

## Abstract

Distinguishing between inflammatory and fibrotic lesions drastically influences treatment decision-making regarding Crohn’s disease. However, it is challenging to distinguish these two phenotypes before surgery. This study investigates the diagnostic yield of shear-wave elastography and computed tomography enterography to distinguish intestinal phenotypes in Crohn’s disease. Thirty-seven patients (mean age, 29.51 ± 11.52; 31 men) were evaluated with average value of shear-wave elastography (Emean) and computed tomography enterography (CTE) scores. The results demonstrated that a positive correlation between the Emean and fibrosis (Spearman’s r = 0.653, *p* = 0.000). The cut-off value for fibrotic lesions was 21.30 KPa (AUC: 0.877, sensitivity: 88.90%, specificity: 89.50%, 95% CI:0.755~0.999, *p* = 0.000). The CTE score showed a positive correlation with inflammation (Spearman’s r = 0.479, *p* = 0.003), and a 4.5-point grading system was the optimal cut-off value for inflammatory lesions (AUC: 0.766, sensitivity: 73.70%, specificity: 77.80%, 95% CI: 0.596~0.936, *p* = 0.006). Combining these two metrics improved the diagnostic performance and specificity (AUC: 0.918, specificity: 94.70%, 95% CI: 0.806~1.000, *p* = 0.000). In conclusion, shear-wave elastography can be used to help detect fibrotic lesions and the computed tomography enterography score emerged as a feasible predictor of inflammatory lesions. The combination of these two imaging techniques is proposed to distinguish intestinal predominant phenotypes.

## 1. Introduction

Crohn’s disease (CD) is a chronic inflammatory disorder that has shown an increasing incidence and prevalence [1,2]. The characteristic transmural inflammation of CD can result in an inflammatory or fibrostenotic phenotype. The assessments of intestinal phenotypes have become a key point in CD patient management. Anti-inflammatory drugs are effective in patients with inflammatory bowel walls [3]. However, effective targeted drugs have not been developed for fibrosis [4]; therefore, endoscopic dilation therapy, or surgical resection are reasonable treatment options [5,6]. Currently, a method of distinguishing inflammation from fibrosis before surgery has not been developed.

In recent years, new cross-sectional imaging techniques that study CD have evolved, and have high accuracy [7]. Ultrasound imaging and computed tomography (CT) have high sensitivity and specificity in the identification of small bowel and colon lesions [8]. Histopathological assessments of inflammation and fibrosis in surgically resected specimens have been performed to validate noninvasive imaging modalities [9]. Research has shown that these two imaging techniques may help distinguish between inflammatory and fibrotic intestines [10]. Ultrasound imaging, a noninvasive method for intestinal lesions, is recommended to be used to detect CD at its first presentation, and to assess CD location, activity and possible complications [11]. A novel ultrasonic imaging technology called shear-wave elastography (SWE) has also evolved. SWE can quantitatively analyze tissue stiffness by measuring shear wave velocity [12]. This technology expands the application of ultrasound to distinguish CD intestinal wall phenotypes, especially real-time two-dimensional shear wave ultrasound elastography (2D-SWE). Compared with traditional strain elastography, 2D-SWE has a lower operational dependence, higher repeatability and relatively objective results [13,14]. Several studies on CD in animal models and human specimens demonstrated that SWE can differentiate between inflammatory and fibrotic lesions [15,16,17]. Computed tomography enterography (CTE) is sensitive to the detection of small bowel disease in CD, and is comparable to magnetic resonance enterography [18]. The studies have found an association between CT findings and inflammatory lesions, including bowel wall thickening, mural hyperenhancement, and comb signs [19,20]. The American College of Radiology has used CTE as an important method for the diagnosis of CD [21]. Several CTE scoring systems have been proposed to study inflammation [22].

The ability of shear-wave elastography and of CT enterography to detect inflammation and fibrosis have not been determined. Moreover, the prior studies have been limited to one of the independent technologies in differentiating intestinal phenotypes. The present study was designed to investigate the diagnostic yield and accuracy of CTE and SWE separately, and the combination of these two techniques, in differentiating inflammatory lesions from fibrotic lesions in CD.

## 2. Materials and Methods

### 2.1. Patients

**Figure 1:**
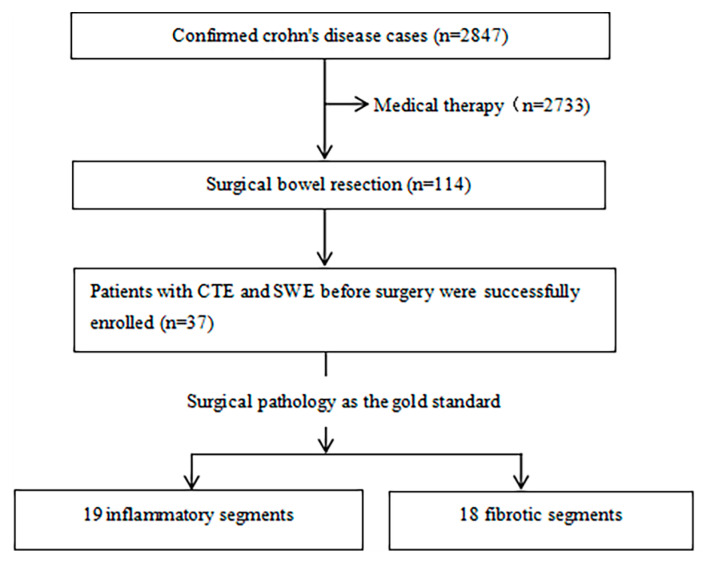
The flow diagram of patients through the study.

We reviewed the radiology and ultrasonic image and electronic medical records for patients with CD from our institution between January 2016 and October 2021. The inclusion criteria were as follows: age > 17 years; terminal ileum lesions; no previous intestinal surgery; and patients who underwent both SWE and CTE examinations within 3 months before surgical resection. The exclusion criteria were as follows: SWE technical failure; and inconsistent segmental positioning of the intestine on ultrasonography, CT and pathology. Figure 1 shows the flow of patients through the study. Of the 114 CD patients with clinical characteristics who underwent bowel surgery, 38 patients underwent both CTE and SWE examinations before surgery. One patient was excluded due to SWE technique failure. The baseline characteristics of the 37 patients with CD are shown in Table 1. Surgical indications included 22 cases of complete intestinal obstruction, 8 cases of fistula and/or abscess formation, and 7 cases of perforation. A histopathologic review of the resected intestines identified 19 inflammatory and 18 fibrotic segments that were finally included for analysis. The number of cases of abscess, fistula, or perforation was greater in inflammatory lesions (inflammation vs fibrosis: 52.63% vs. 28%), and obstruction was more common in fibrotic lesions (inflammation vs fibrosis: 47.00% vs. 72.72%).

### 2.2. Traditional Intestinal Ultrasound and 2D-SWE Imaging

All transabdominal ultrasounds were performed by ultrasonographers who had more than 10 years of experience in bowel ultrasound. Patients were asked to fast for a period of at least 8 h before the ultrasound examinations, which were performed using an Aixplorer US system (SuperSonic Imagine, Aix-en-Provence, France). A convex broadband probe SC6-1 (3.5~5.5 MHz) was used to observe the whole intestine and locate the lesion. A linear array probe SL15-4 (4~15 MHz) was then used to evaluate the bowel wall thickness and blood flow patterns. Limberg grading [23] of bowel vascularization in the lesions was recorded by color doppler imaging: grade I, bowel wall thickening (>3 mm) without vascularization; grade II, bowel wall thickening with spot vascular signals; grade III, bowel wall thickening with longer vascular signals; and grade IV, bowel wall thickening with longer vascular signals extending from the mesentery. In the longitudinal section of intestinal segments, the probe was fixed in position and then switched to SWE mode, with Young’s modulus set in kPa. In addition, the patients were required to breath hold for 3~5 s. After the elastogram was uniform and stable, the frame was fixed and stored. The Q-box region of interest was placed in the elastogram to measure Emean (the average values of SWE) with 3 times, and averaged values were used for final data analysis.

### 2.3. CT Enterographic Techniques

**Figure 2:**
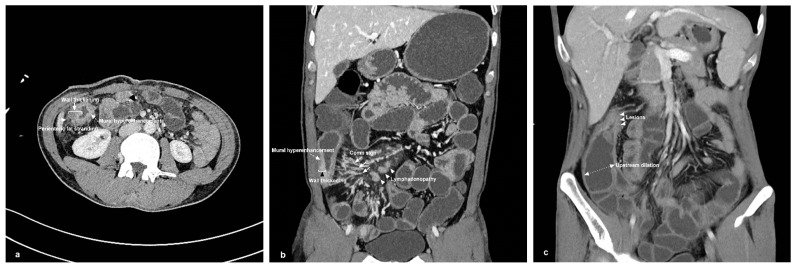
CT enterography findings in Crohn’s disease. (**a**) A male, 29 years old. Axial CT enterography in a patient with Crohn’s disease shows bowel wall thickening (10.9 mm), mural hyperenhancement and perienteric fat stranding (arrows); (**b**) Coronal CT image shows the mesenteric straight small vessels are increased, presenting as comb sign. The enlarged mesenteric lymphadenopathy, bowel wall thickening, and mural hyperenhancement also can be clearly seen (arrows); (**c**) A male, 41 years old. Coronal CT image shows the lesion and upstream dilation (39.7 mm) (arrows).

Patients were asked to fast for a period of at least 8 h before the CT scan. Intestinal cleansing was performed by oral administration of a 1500 mL compound polyethylene glycol electrolyte powder solution (Wang Pharmaceutical Co., Ltd., Shenzhen, China) four hours prior to the scan. One hour prior to the scan, 2000 mL of 2.5% isotonic mannitol (administered in 4 doses of 500 mL/time) was given orally to maintain the filled state of the small intestine [24]. After the third 500 mL dose (30 min before the scan), the patient was administered an intramuscular injection of anisodamine 10 mg to promote and maintain gastrointestinal muscle relaxation. The remaining 500 mL was administered before examination. CT scanning ranged from the top of the diaphragm to the pubic symphysis using dual-source CT (Siemens Somatom Force system, Erlangen, Germany). One hundred milliliters of nonionic contrast agent was injected intravenously at 4 mL/s (Lopamiro370, Bracco S.P. A, Shanghai, China), and 50 mL of normal saline was added at the same flow rate to obtain enhanced CT images. By adopting advanced modeled iterative reconstruction, the fast double energy method was used to reconstruct 5 mm layer thicknesses, and a layer spacing of 5 mm was used for daily imaging of abdominal window images. Reconstruction of a 1~1.5 mm window and incremental reconstruction of a 0.7~1 mm thin abdominal soft tissue window were performed and delivered to the workstation. Coronal and sagittal images were reconstructed on the workstation to display the lesion and its surrounding tissues in multiple directions. Radiologists with more than 10 years of experience in abdominal CT who were blinded to both the clinical history and the pathologic findings retrospectively reviewed all CT scans. The CTE findings that were scored included mural hyperenhancement, bowel wall thickening (>3 mm), comb sign, perienteric fat stranding, upstream dilation (small bowel ≥ 30 mm, colon > 60 mm), and lymphadenopathy (short-axis diameter >15 mm) (Figure 2) [25]. Each of these CTE findings was scored 0 or 1. The total CTE scoring was the accumulation of each score (range: 0~6).

### 2.4. Histological Evaluation

The surgical specimens were examined and processed in a standard routine clinical protocol, including tissue fixation in formalin and paraffin-embedded intestinal tissues. Hematoxylin and eosin [H and E]-stained sections were selected for the histological analysis. For each layer of the bowel wall, the possibility of histological abnormalities was assessed individually by a pathologist who was blinded to the results of other examinations. The assessment of inflammation included neutrophil infiltrate, cryptitis, crypt abscess, erosion, and ulcer, while that of fibrosis included hyperplasia of fibrous tissue and hypertrophic nerves [10,26,27]. In the present study, the basic criterion for determining inflammatory segments was inflammation without fibrosis in any layer. Fibrotic segments were defined as pronounced areas of submucosal fibrosis or hypertrophic nerves.

### 2.5. Statistical Analysis

The Kolmogorov–Smirnov test was applied to test for a normal distribution. For a normal distribution, continuous variables were presented as the mean ± SD and T tests were used. For nonnormal distributions, the Mann–Whitney U test was used. Categorical variables were tested by the chi-square test. The Spearman product moment correlation coefficient was used to determine the relationship between the CTE score or Emean and intestinal wall phenotypes. Receiver operating characteristic (ROC) curves were used to assess the diagnostic yield, sensitivity, and specificity of the CTE score, Emean, and the combination of these two metrics to distinguish intestinal phenotypes in CD. Statistical analyses were performed using IBM SPSS 19.0 software (SPSS Inc., Chicago, IL, USA). The results were considered significant at a *p* value less than 0.05. Graphing was performed using GraphPad Prism 6.0. The statistical review of the study was performed by a biomedical statistician.

## 3. Results

### 3.1. Conventional Ultrasound and Two-Dimensional Shear-Wave Elastography (2D-SWE)

**Figure 3:**
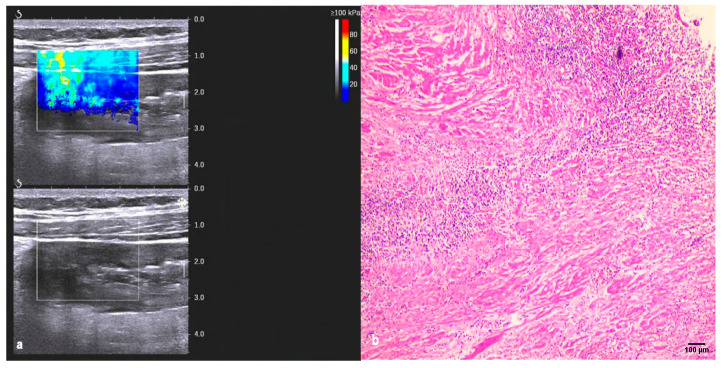

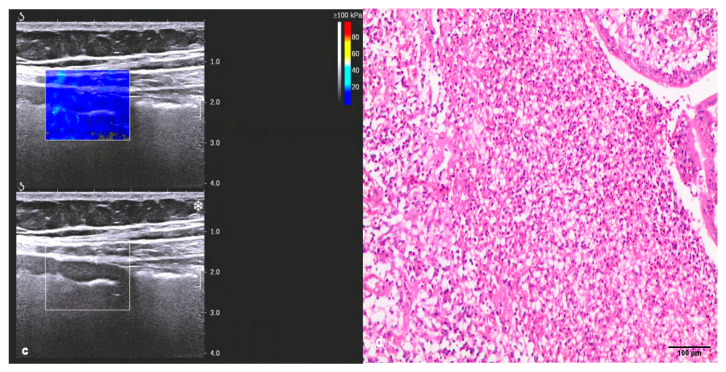
Real-time shear-wave elastography and histopathology of intestinal wall in patients with CD. (**a**) A male, 29 years old, Emean = 54.9 Kpa; (**b**) Haematoxylin and eosin [H and E]-stained sections of the same patient; the histological findings of intestinal wall showed the areas of submucosal fiber hyperplasia were pronounced; (**c**) A female, 55 years old, Emean = 13.0 Kpa; (**d**) Haematoxylin and eosin [H and E]-stained sections; the histologically confirmed inflammatory lesion.

Increased bowel wall thickening at multiple segments with a maximum thickness ranging 4.00~21.00 mm and an average thickness of 9.80 ± 3.91 mm was observed. The study also compared the Doppler US of mesenteric blood flow. The analysis of the data revealed no difference in intestinal wall thickness or Limberg grading between the inflammatory and fibrotic bowel segments (*p* > 0.05). However, the Emean was notably higher for fibrotic bowel segments than inflammatory segments (*p* < 0.05) (Figure 3, Table 2). A positive correlation was observed between the Emean and fibrosis (Spearman’s r = 0.653, *p* = 0.000). To assess the role of the Emean in distinguishing inflammatory from fibrotic segments, pathological tissue was used as the gold standard. The results indicated that Emean > 21.3 kPa could be used for fibrotic lesions. The sensitivity and specificity were 88.90% and 89.50%, respectively, with an AUC of 0.877 (95% CI: 0.755~0.999, *p* = 0.000).

### 3.2. CTE Findings and Diagnostic Yield of CD Phenotypes

**Figure 4:**
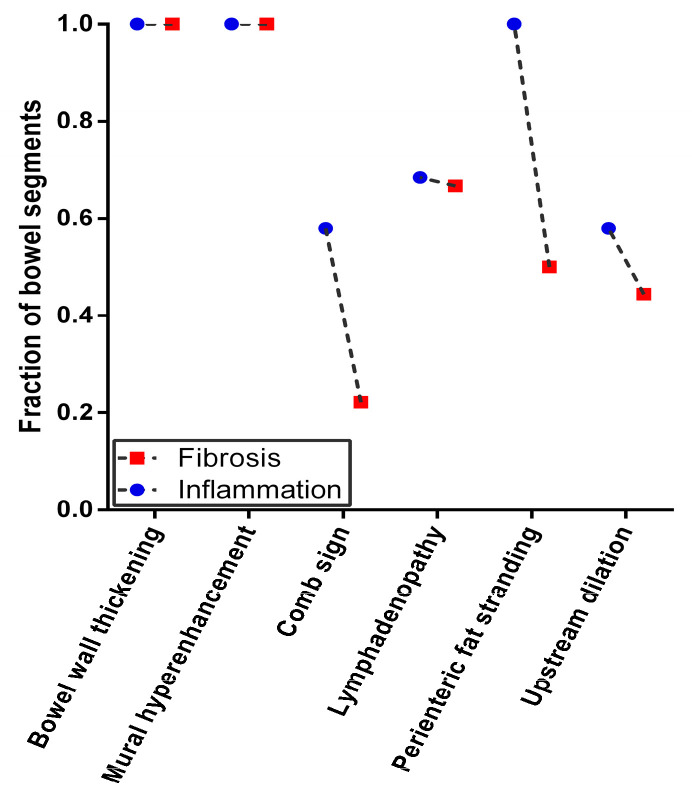
CT enterography findings to differentiate inflammation from fibrotic segments in Crohn’s disease. The observation to emerge from the CT findings showed comb sign and perienteric fat stranding were notably higher for inflammatory segments versus fibrous bowel segments.

The single most striking observations to emerge from the CT findings comparison were comb signs and perienteric fat stranding between the inflammatory and fibrotic bowel segments (*p* < 0.05) (Table 2, Figure 4). Moreover, the CTE score was calculated based on careful evaluation of the CTE findings in each bowel segment. The CTE score was notably higher for inflammatory segments versus fibrotic bowel segments (Table 2). A positive correlation was observed between the CTE score and pathology findings with regard to inflammation (Spearman’s r = 0.479, *p* = 0.003). Furthermore, an ROC curve was generated to assess the role of the CTE score in CD. Using a 4.5-point grading system for inflammatory lesions, the sensitivity and specificity of the CTE score were 73.70% and 77.80%, respectively, with an AUC of 0.766 (95% CI: 0.596~0.936, *p* = 0.006).

### 3.3. CTE Combined with 2D-SWE to Distinguish between Inflammatory and Fibrotic Lesions

**Figure 5:**
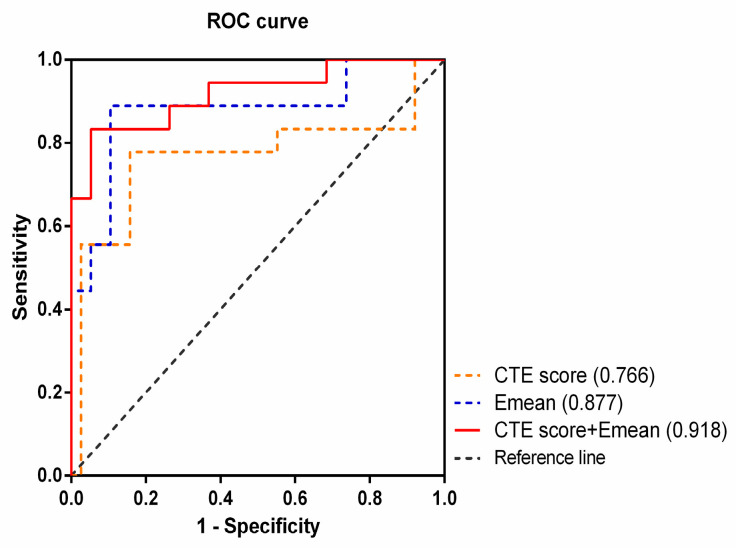
The metrics in discriminating between inflammation and fibrosis lesions. The ROC curve shows the combination of CT enterography score (CTE) and the average value of Shear-wave elastography (Emean) is superior to either one alone in discriminating between inflammation and fibrosis lesions in Crohn’s disease.

Based on the predictor identified in this study for inflammatory and fibrotic lesions of CD, the yield and accuracy of the combination of CTE score and Emean in distinguishing inflammation from fibrotic lesions were determined. Interestingly, when these two metrics were combined to discriminate between inflammation and fibrotic lesions, the sensitivity and specificity were 83.30% and 94.70%, respectively, with an AUC of 0.918 (95% CI: 0.806~1.000, *p* = 0.000) (Figure 5).

## 4. Discussion

The distinction between inflammation and fibrotic lesions drastically influences treatment decision-making regarding Crohn’s disease. Patients with prominent inflammation can potentially be managed with medical agents and biological therapies, whereas patients with prevalent fibrosis, in particular if associated with obstructive symptoms, frequently require endoscopic balloon dilatation or surgery [3,28,29]. However, both types had been resected in this study. It is hoped that this differentiation will be valuable in the clinical field, highlighting the need for an accurate and noninvasive method of characterizing intestinal phenotypes before surgery. Cross-sectional imaging techniques such as shear-wave elastography and CT enterography are suited for this task. In this present study, the two methods were both used in the assessment, and their diagnostic accuracies were compared. The average values of the SWE (Emean) and CT enterography scores showed excellent diagnostic yield for fibrotic or inflammatory lesions, respectively. Although the sensitivity of the combination of these two metrics tended to be lower than that of the Emean, the specificity was outstanding in differentiating intestinal phenotypes in Crohn’s disease.

To distinguish intestinal inflammation and fibrotic lesions, the direct detection of intestinal wall tissue should be the most intuitive and reliable method. 2D-SWE provides a real-time elastogram of the inflammation and fibrous intestinal segment regions, and can perform quantitative detection of intestinal wall stiffness. The Emean were higher for fibrous bowel segments versus inflammatory segments, which is consistent with previous studies [15,30]. Mesenchymal cells, such as fibroblasts, myofibroblasts, and smooth muscle cells, increase in fibrotic intestinal segments, resulting in the accumulation of a collagen-rich extracellular matrix [31]. The extracellular matrix is a storage site for fibrotic growth factors [32,33], which can be released to promote the deposition and cross-linking of extracellular matrix components, thereby changing the local mechanical properties and increasing the stiffness [34]. In addition, the contraction of mesenchymal cells can further increase stiffness, independent of inflammation [35]. In clinical practice, intestinal wall fibrosis is a serious issue. Among the predictors for fibrosis intestinal segments, the Emean value was prominent. These findings demonstrate the reliability of the SWE technique in assessing intestinal wall fibrosis, but not inflammatory lesions [36]. Regarding the reference value of fibrosis, liver ultrasound elasticity guidelines recommend using the mean value for liver stiffness assessments [14]. This value was also applied in our study. The present study revealed that using 21.30 KPa as the cutoff value could discriminate between inflammation and fibrosis, and showed excellent sensitivity. Although the specificity tended to be lower than that of prior studies that used 22.55 kPa to discriminate between mild/moderate and severe fibrosis [16], this difference was not significant (89.5% vs. 91.7%). Notably, this study only distinguished between inflammation and fibrotic lesions and did not grade the severity of fibrosis. Therefore, regarding the accuracy of the threshold, a larger sample should be included and further study is warranted in the future. CTE is a novel and potentially useful radiological technique for evaluating the intestine in CD, and has a high accuracy in detecting active inflammation [37]. A previous study showed that CTE may reliably differentiate between inflammatory and fibrotic lesions in patients with CD [26]. In addition, several CTE variables were associated with the pathological findings of inflammation, including mucosal enhancement, comb signs, and perienteric fat stranding can predict tissue inflammation [27]. A prior study noted the importance of perienteric fat in the pathogenesis of inflammatory and fibrotic lesions in CD [38]. Indeed, in the present study, the comb sign was prominent in inflammatory bowel segments, but only 22.22% in fibrotic bowel segments. Perienteric fat stranding was also found to be associated with characteristics of the intestinal wall phenotype. When the bowel is in active inflammation, a series of inflammatory reactions may occur around the diseased bowel. Intestinal ulcers associated with CD are primarily located at the mesenteric margin, and increased TNF-α production by fat cells in mesenteric fat may contribute to transmural inflammation [38]. To some extent, CTE reflects the histopathological state of CD inflammation. However, in this study, mural hyperenhancement was found in all intestinal segments. In fact, pure fibrotic lesions in CD are rare, and inflammation and fibrosis often coexist [27]. The establishment of the CTE scoring system provides an objective tool for disease assessment [39]. The CTE score represents an accumulation of intestinal CT findings. The present study implies that the CTE score represents a reliable predictor of inflammatory lesions, but has little predictive value for tissue fibrosis lesions, which confirms the view of a previous study [27]. For a threshold of predicting inflammatory lesions of 4.5 points, the sensitivity and specificity was 73.70% and 77.80%, respectively. However, a prior study indicated that a threshold of 3.5 points has a sensitivity of 88% and a specificity of 86% for active inflammation [40]. The difference between the results is related to the CT findings and the number of reference indicators included in the CTE scoring system. In the present study, the total score range was 0 to 6, while that in the previous study was 0 to 5. Prestenotic dilation and lymphadenopathy were included in the study, while fat in the layers of the bowel wall was absent. The other four CTE findings were the same. Therefore, the application scoring value varies with the scoring system. Our study revealed the role of SWE in detecting fibrotic bowel segments and CTE in evaluating CD inflammatory lesions, and investigated the combination of the two imaging techniques. Interestingly, a finding to emerge from this study is that the combination of SWE with CTE contributed to a better diagnostic performance in distinguishing inflammation from fibrotic lesions, and the specificity was significantly improved. Although SWE and CTE have their own shortcomings, by combining the two technologies to assess the intestinal wall phenotypes of CD, they can compensate for each other’s shortcomings, and the advantages are more prominent.

This study had several potential limitations. This makes intuitive sense given that blood flow correlates with active inflammation. Despite the fact that there is a combination of active inflammation and fibrotic lesions in the intestine, we expect to be able to better distinguish which phenotype is predominant in intestinal lesions. The comprehensive assessment of CTE and SWE is more suitable for this task, and contributes to optimizing care for patients with CD. In addition, there is a selection bias. Patients who had resection and two types of imaging were retrospectively selected. These are preliminary results from a small study, so larger prospective studies are needed for verification. Finally, the same morphologic changes also vary by different imaging techniques, and challenges remain regarding differentiating inflammation and fibrosis.

## 5. Conclusions

Shear-wave elastography helps detect intestinal fibrosis lesions, and the CT enterography score represents a feasible and accurate predictor of inflammatory lesions in Crohn’s disease. Moreover, combining shear-wave elastography and the CT enterography score can more reliably differentiate intestinal predominant phenotypes in Crohn’s disease. Either technique or a combination of one with intestinal ultrasound is important. Further validation on a wide-scale population is needed.

## Figures and Tables

**Table 1:** The demographics characteristics in patients with Crohn’s disease.

Parameters	Value
Age, Mean (SD), Years	29.51 (11.52)
Gender, M/F (n)	31/6
Clinical characteristics	
Lower abdominal pain	37
Diarrhea	22
Abdominal mass	16
Weight loss	11
Fever	7
Hematochezia	10
White blood cell (10^9^/L)	7.89 ± 3.89
Hemoglobin (g/L)	109.84 ± 22.25
Platelet (10^9^/L)	349.73 ± 122.64
Neutrophil (10^9^/L)	6.53 ± 4.00
Lymphocyte (10^9^/L)	0.96 ± 0.63
Total protein (g/L)	59.16 ± 9.41
Albumin (g/L)	31.38 ± 8.58
Erythrocyte sedimentation rate (μg/g)	33.70 ± 20.62
C-reactive protein (mg/L)	53.42 ± 39.10

**Table 2:** Ultrasound and CTE findings in differentiating intestinal inflammation and fibrosis lesions.

	Inflammation	Fibrosis	*p*-Value
Bowel wall thickness (mm)	9.07 ± 4.14	10.53 ± 3.66	NS
Limberg grading, n (%)			NS
I	0 (0.00)	1 (5.56)	
Ⅱ	2 (10.53)	8 (44.44)	
Ⅲ	12 (63.16)	7 (38.89)	
Ⅳ	5 (26.32)	2 (11.11)	
2D-SWE, Emean, Kpa	17.59 ± 7.21	35.24 ± 13.31	0.000 *
CTE findings, n (%)			
Bowel wall thickening	19 (100.00)	18 (100.00)	NS
Mural hyperenhancement	19 (100.00)	18 (100.00)	NS
Comb sign	11 (57.89)	4 (22.22)	0.020 *
Lymphadenopathy	13 (68.42)	12 (66.67)	NS
Perienteric fat stranding	19 (100)	9 (50.00)	0.000 *
Upstream dilation	11 (57.89)	8 (44.44)	NS
CTE score	4.84 ± 0.77	3.83 ± 1.15	0.004 *

2D-SWE: two-dimensional shear wave ultrasound elastography; CTE: computed tomography enterography; *: *p* < 0.05, significance; NS: *p* > 0.05, no significance.

## Data Availability

The data that support the findings of this study are available from the corresponding author upon reasonable request.
